# Supplementary material for: A Bayesian model for unsupervised detection of RNA splicing based subtypes in cancers
Source: Nat Commun. 2023 Jan 4;14:63. doi: 10.1038/s41467-022-35369-0 (PMC9813260; doi:10.1038/s41467-022-35369-0)
Supplement: Supplementary file 1 — Supplementary Information [file 41467_2022_35369_MOESM1_ESM.pdf]

A Bayesian model for unsupervised detection of RNA splicing based subtypes in cancers

David Wang,[1,2] Mathieu Quesnel-Vallieres,[1,3] Paul Jewell,[1] Moein Elzubeir,[1] Kristen Lynch,[1,3] Andrei Thomas-Tikhonenko,[4,5] Yoseph Barash[1,6*]

[1]Department of Genetics, Perelman School of Medicine, University of Pennsylvania
[2]Graduate Group in Genomics and Computational Biology, Perelman School of Medicine, University of Pennsylvania
[3]Department of Biochemistry and Biophysics, Perelman School of Medicine, University of Pennsylvania
[4]Department of Pathology and Laboratory Medicine, Perelman School of Medicine, University of Pennsylvania
[5]Division of Cancer Pathobiology, Children's Hospital of Philadelphia
[6]Department of Computer and Information Sciences, School of Engineering, University of Pennsylvania

*To whom correspondence should be addressed; E-mail: yosephb@upenn.edu.

Contents

Supplementary Notes 4

1 Data Description **4**

Supplementary Note 1.1: Datasets 4

Supplementary Note 1.2: Splicing Event Definitions 6

2 Additional Methods and Evaluation **7**

Supplementary Note 2.1: Parametric Bootstrap Kolmogorov-Smirnov Test 7

Supplementary Note 2.2: Statistical Methodology for Model Evaluations 8

Supplementary Note 2.3: Generative Synthetic Data Simulations 10

Supplementary Note 2.4: Infinite Mixture Behavior Evaluation 11

Supplementary Note 2.5: Runtime and Memory Evaluation 12

3 CHESSBOARD Pipeline and Features **13**

Supplementary Note 3.1: Standard CHESSBOARD Pipeline for Real Data Analysis . 13

Supplementary Note 3.2: Convergence Diagnostics 14

Supplementary Note 3.3: Using CHESSBOARD as a Predictive Model 17

Supplementary Note 3.4: Statistical Testing in Regulation Analysis with ENCODE Data 17

Supplementary Note 3.5: CHESSBOARD Can Rank Tile's Splicing Events for Downstream Analysis 19

4 beatAML Analysis **20**

Supplementary Note 4.1: Recursive Clustering and Termination 20

Supplementary Note 4.2: Multiple Testing Correction with Missing Data 22

5 Drug Response Analysis **23**

Supplementary Note 5.1: Drug Response Correlation 23

Supplementary Note 5.2: Variance Explained 24

Supplementary Note 5.3: Decision Tree Permutation Test 25

6 Model Details **26**

Supplementary Note 6.1: Variable Table 26

7 Survival Analysis **27**

Supplementary Note 7.1: Survival Analaysis 27

Supplementary Figures **28**

Supplementary Fig. 1: Additional Model Evaluation 28

Supplementary Fig. 2: Runtime and Memory Evaluation. 30

Supplementary Fig. 3: MCMC Convergence Evaluation. 33

Supplementary Fig. 4: Additional beatAML Data Analysis. 35

Supplementary Fig. 5: Gene Ranking Analysis. 36

Supplementary Fig. 6: Additional Drug Response Analysis. 37

Supplementary Fig. 7: Survival Analysis. 39

Supplementary Data **41**

Supplementary Data 1: beatAML 41

Supplementary Data 2: beatAML Recursive Step 1 41

Supplementary Data 3: beatAML AML Genes 41

Supplementary Data 4: Drug p-values 41

Supplementary Data 5: TARGET AML 41

Supplementary Data 6: TARGET B-ALL 41

Supplementary Notes

1 Data Description

Supplementary Note 1.1: Datasets

beatAML Dataset

The beatAML data used in this study includes RNA-seq data from 451 specimens from 411 patients from Tyner et al. 2018 and an additional 26 samples for a total of 477 samples[1]. The samples were sequenced from purified mononuclear cells collected from peripheral blood or bone marrow. All patients were diagnosed with AML or closely related disease. We downloaded FASTQ files from www.synapse.org to use in subsequent processing and analysis. Sequencing adaptors and low quality base calls were trimmed from FASTQs using trim galore. For expression data, we obtained TPM values using SALMON with Hg38 decoys from the FASTQ files. For splicing data, we aligned the FASTQs using STAR and sorted the BAM files using samtools. We mapped reads in the BAM files to splice junctions using MAJIQ using ensembl GRCh38 v94 annotations. The build contained all the beatAML, TARGET pediatric AML and TARGET B-ALL samples such that all 3 datasets use the same splice graph.

beatAML Drug Sensitivity Data

The drug sensitivity data was taken directly from the supplied beatAML metadata. The IC_{50} values were generated using an ex vivo drug sensitivity assay described in Tyner el. al 2018[1]. The screen applied 122 small molecule inhibitors to isolated mononuclear cells from the AML patient samples. The IC_{50} value for each drug-sample pair was calculated by fitting a sigmoid curve to 7 data points representing cell viability at varying drug concentrations and estimating the concentration that resulted in 50% viability. Note that the drug concentrations were measured using 3-fold serial dilution in the range of 10uM to 0.0137 uM. Many drugs-

sample pairs had an IC_{50} of 10uM indicating that a wider range of concentrations were needed to properly fit the curve. In such cases, measuring the area under the curve (AUC) provides a better representation of drug sensitivity.

beatAML Mutation Data

All mutation annotations for beatAML samples used in this study were taken directly from the supplied metadata. The consensus variant calls were generated from multiple genotype callers applied to whole exome sequencing (WES) data and assigned a mutation status based on ensembl VEP GRCh37 annotations as described in Tyner et. al 2018[1]. *FLT3*-ITD, *NPM1* and *CEBPA* mutations for a subset of patient sample were verified using additional experimental assays.

Penn HTSC Dataset

The Penn HTSC dataset contains 77 AML patient samples sequenced by the University of Pennsylvania high-throughput screening core. All samples used in the sequencing were confirmed to be at least 90% AML blasts. A subset of the samples (29) was previously published in Rivera et al. 2021[2]. This data can be obtained from GEO (GSE142514).

ENCODE Knockout Dataset

The ENCODE knockout dataset was taken from Van Nostrand et al. 2020 and processed as described in Slaff et al. 2021[3;4]. The subset of data we used in this study was limited to knockout experiments of 106 RBPs/SFs in K562 cell lines that had matching eCLIP data. This data was generated in 32 batches with each batch containing at least 2 replicates for controls and knockout experiments. The batch effect was corrected across the knockout experiments using MOCCASIN as described in Slaff et al. 2021[4]. For differential splicing analysis, controls across the batches were aggregated into “virtual controls” and compared against each knockout experiment using MAJIQ.

ENCODE eCLIP Dataset

The data was downloaded from www.encodeproject.org. We only used data for which there was a matching RBP/SF knockout experiment (106 RBPs/SFs) in K562 cell lines. Each RBP/SF experiment had 2 replicates. Consensus binding peak calls were obtained using irreproducible discovery rate (IDR) (https://www.encodeproject.org/data-standards/terms/#concordance).

TARGET B-ALL

The TARGET B-ALL data used in this study includes 517 RNA-seq samples from 250 unique patients with ALL diagnosis. Most patients are represented by samples taken at primary leukemia diagnosis and samples taken after relapse with 2-3 replicates. Only samples with annotated B-cell origin or inferred B-cell origin were considered. The inferred cell origin labels were taken from Slaff et al. 2021 which annotated the cell origin of unannotated samples based on the cell origin of the samples they clustered with[4]. Batch effects were corrected using MOCCASIN for sequencing instrument (HiSeq 2500 vs HiSeq 2000).

TARGET Pediatric AML

The TARGET Pediatric AML data was downloaded from https://portal.gdc.cancer.gov. We selected only samples with an age under 23. The curated dataset contained 612 samples.

Supplementary Note 1.2: Splicing Event Definitions

The input to CHESSBOARD is a data matrix $X_{n \times m}$ with n columns representing patient samples and m rows representing splicing events. The definition of what constitutes an alternative splicing event may vary depending on the quantification tool the chosen by the user. Regardless of the tool, a user can supply two TSV files where each row is a splicing event. The entries in the first file represent reads mapped to the junction of interest while the entries in the second file represent the sum of all reads not mapped to this junction of interest but are still contained in the splicing event/normalization unit. For example, if a user chooses to quantify 'classical'

events such as cassette exons using a method such as rMATS[5], they can parse rMATS output such that the input files reports inclusion reads vs the total exclusion reads per AS event. In terms of CHESSBOARD's model, the reads mapped to a cassette exon's inclusion junction are the successes x_{ij} and the total reads mapped to the AS event (in this example exclusion reads plus inclusion reads) are η_{ij}.

In this study, we use MAJIQ[6] which defines AS events using the concept of local splice variations (LSVs). Briefly, Each LSV has a reference exon to which other exons (or introns, for intron retention events) are spliced to. A source LSV contains a set of splice junctions downstream of the reference exon while a target LSV contains a reference exon that is spliced to other exons or introns upstream of it. MAJIQ's LSV formulation is able to capture classic event types such as cassette exons, but also many other splicing variations that are more complex (involve more that two alternative junctions) as well as unannotated junctions and exons. Within an LSV, a splice junction is quantified by percent splice in (Ψ) which is the ratio of reads spanning the junction to all other reads in the LSV. However, CHESSBOARD's binomial model is designed to handle one junction per splicing event. Thus during the processing of MAJIQ's output, we select as a representative junction per LSV. This junction is determined as the one in the LSV with the highest variance in Ψ across all samples. We note that CHESSBOARD is able to support any representation of splicing as input as long as it can be expressed as ratios of reads. This includes isoform ratios although this is not recommended due to complications with resolving isoform abundance using only short read RNA sequencing.

2 Additional Methods and Evaluation

Supplementary Note 2.1: Parametric Bootstrap Kolmogorov-Smirnov Test

Part of CHESSBOARD's prefiltering pipeline involves removing non-informative LSVs which only exhibit a single Ψ modality using a parametric bootstrap Kolmogorov-Smirnov (PBKS)

test. CHESSBOARD assumes that the Ψ distribution of each LSV is a mixture of 2 Beta distributions representing a signal and background component thus LSVs that can be modeled by a single distribution are unlikely to contain any signal with notable effect size. The null hypothesis (H0) of the PBKS test is that the data is Beta distributed. The alternate hypothesis (H1) is that the data is not Beta distributed. Rejecting H0 would suggest that a single component Beta is a poor fit for the data and thus multiple modalities exist. We first fit a Beta distribution to the Ψ values for a given LSV using MLE. We then compute the 2 sided 1 sample KS statistic D* using the fitted Beta distribution as the reference

$$D* = max(\hat{F}(x) - G(x)) \tag{1}$$

$\hat{F}$ is the empirical CDF and G is the CDF of the fitted Beta. Note that because, the reference distribution was obtained from the data, D* no longer follows the Kolmogorov distribution. Thus we used a parametric bootstrap approach to estimate the p-value. First, we sample n datasets of equal size to the empirical data from the fitted Beta distribution where n is the number of user defined bootstrap samples (default = 500). We then compute D* on each dataset. The PBKS test p-value is equal to the proportion of bootstrapped D* statistics that are greater than the observed D* statistic.

Supplementary Note 2.2: Statistical Methodology for Model Evaluations

Information Gain

Information gain (IG) can be interpreted as the amount of information gained by a random variable given that a dependent variable is observed. The quantity is related to Kullback-Leibler divergence (KLD) in that IG = 0 when the KLD between the joint distribution and product of marginals = 0 (i.e. variables are independent). In a k = 2 clustering setting, IG can be interpreted as the amount of information gained by performing/observing a split of the population into two

groups. We compute IG as follows

$$1 - \sum_{k}^{2} -\frac{I(\hat{c}_i = k)}{N}[P(c_i = 2|\hat{c}_i = k)log_2(P(c_i = 2|\hat{c}_i = k))+$$
$$P(c_i = 1|\hat{c}_i = k)log_2(P(c_i = 1|\hat{c}_i = k))] \quad (2)$$

The population entropy in our experimental setting is 1 since the 2 classes have equal size. The second term represents a weighted average of the clustering entropy given observation of an inferred binary label for each sample $\hat{c}_i$.

Precision and Recall Based on Recovery and Relevance Score Precision τ_{pr} and recall τ_{rc} metrics based on Recovery Relevancy Score are given by

$$\tau_{pr} = \frac{\sum_{(G_1,C_1)\in M_1} max_{(G_2,C_2)} \frac{|G_1 \cap G_2| + |C_1 \cap C_2|}{|G_1| + |C_1|}}{|M_1|}$$
$$\tau_{rc} = \frac{\sum_{(G_1,C_1)\in M_1} max_{(G_2,C_2)} \frac{|G_1 \cap G_2| + |C_1 \cap C_2|}{|G_2| + |C_2|}}{|M_1|} \quad (3)$$

Here, M_1 represents a set of reference tiles and each tile is defined by a feature set G_1 and sample set C_1. M_2 represents the set of inferred tiles where each tile is defined by a feature set G_2 and sample set C_2. For each tile in the reference set, select 1 tile in the inferred set which best represents this tile based on the maximum precision or recall of the inferred feature and sample sets. Compute this quantity of each reference tile and average the quantities.

Adjusted Rand Index

Adjusted Rand Index is a measure of clustering agreement. A value of 1 indicates perfect agreement. The score is computed as

$$ARI = \frac{\sum_{ij}(\binom{n_{ij}}{2} - [\sum_i \binom{a_i}{2} \sum_j \binom{b_j}{2})]/\binom{n}{2}}{\frac{1}{2}[\sum_i \binom{a_i}{2} + \sum_j \binom{b_j}{2}] - [\sum_i \binom{a_i}{2} \sum_j \binom{b_j}{2})]} \quad (4)$$

Here, n_{ij} represents the number of samples assigned to group i in the first clustering and group j in the second clustering. $a_i = \sum_j n_{ij}$ and $b_j = \sum_i n_{ij}$.

Effective Dimensionality

Effective dimensionality represents the strength of the concentration parameter as a function of the number of features. Intuitively, as the number of features increases, the impact of the concentration parameter decreases since the total likelihood of a sample compounds multiplicatively with the number of features. Therefore a smaller concentration is needed as the feature space increases to maintain constant effect. We define effective dimensionality as

$$\alpha_d = \frac{\alpha}{N - 1 + \alpha}^{d} \tag{5}$$

where α_d is the concentration parameter chosen to have the same effect as α when the effective feature set size is d and N is the number of samples. This reduces to the standard CRP prior for opening a new cluster in a 1 dimensional setting (i.e d = 1).

Supplementary Note 2.3: Generative Synthetic Data Simulations

To generate synthetic data for testing our model, we first simulated a background matrix with 100 samples (columns) and 100 LSVs (rows). For each LSV j and sample i, we simulated the read rate x_{ij} from the following generative process:

$$\begin{aligned} x_{ij} &\sim Binomial(n_j, \Psi_j^{BG}) \\ \Psi^{BG} &\sim Beta(\alpha_j^{BG}, \beta_j^{BG}) \\ n_j &\sim Poisson(\lambda_j) \end{aligned} \tag{6}$$

We then implanted tiles with read rates simulated from the following generative process:

$$\begin{aligned} x_{ij} &\sim Binomial(n_j, \Psi_j^{S}) \\ \Psi^{S} &\sim Beta(\alpha_j^{S}, \beta_j^{S}) \\ n_j &\sim Poisson(\lambda_j) \end{aligned} \tag{7}$$

Here $Beta(\alpha_j^{BG}, \beta_j^{BG})$ represents the background Ψ distribution, $Beta(\alpha_j^{S}, \beta_j^{S})$ represents the signal Ψ distribution and $Poisson(\lambda_j)$ represents the distribution over read coverage or expres-

sion level for a LSV. Poisson modeling for gene expression has been used in several methods because read dispersion tends to increase with expression levels. The parameters of the simulation were estimated from the beatAML dataset. To estimate α and β, we fit a 2 component Beta mixture to the Ψ values of the 1000 most variable LSVs. The estimated parameters for the component with lower variance were used for the background. To estimate λ, we use the median read rate for each LSV. We selected a random subset of 100 LSVs with $|E(\Psi^{BG}) - E(\Psi^{S})| > 0.2$ to parameterize each LSV in the simulation.

Supplementary Note 2.4: Infinite Mixture Behavior Evaluation

CHESSBOARD is able to learn the number of tiles without a priori knowledge using an infinite mixture modeling approach with a Chinese Restaurant Process (CRP) prior (Methods). This can be particularly effective for analyzing cancer data where the number and size of disease subtypes is unknown. Specifically, CHESSBOARD naturally models both common and rare subtypes since the CRP prior expects a gradient of group sizes. However, like other unsupervised methods, CHESSBOARD depends on both hyperparmeters and characteristics of the data. Here, we show how the CRP concentration hyperparameter, which regularizes the number of tiles, interacts with two characteristics of the data: The number of LSVs supporting a tile and the signal strength within an LSV measured by Kullback Leibler (KL) divergence between the signal and background distributions. In addition, we evaluate the algorithm's ability to identify tiles under two different scenarios: One where it must assign samples to the correct tile such as when the clusters are initialized using k-means and another when it must overcome the concentration parameter to create a new tile. The results of these evaluations are summarized in Fig. 2e. As expected, we find that as the distance between the signal and background distributions increases, fewer supporting LSVs are needed to assign a sample to the tile with high (> 0.99) probability, ranging from 1 for KL = 6.697 to 10 for KL = 0.617 (Supplementary Fig.

1a left). We also find that learning new clusters requires far more supporting LSVs or a much stronger signal-background discrepancy due to the penalty of opening a new cluster induced by the concentration parameter. By setting the concentration parameter using effective dimensionality (Supplementary Note 2.2), we show that it takes at least 20 supporting LSVs to find a new cluster when the KL divergence is high (Supplementary Fig. 1a right). Finally, we evaluate whether these observations hold on more realistic synthetic data by simulating a matrix with 2 tiles where the average $\Delta\Psi$ between signal and background distributions is 0.2, a threshold commonly used in the RNA field to define a significant splicing change. We then add a 3rd tile with a varying number of supporting LSVs. We find such data requires at least 12 supporting LSVs to find the 3rd tile (Supplementary Fig. 1b).

Supplementary Note 2.5: Runtime and Memory Evaluation

To assess the runtime and memory usage of CHESSBOARD, we ran the algorithm on synthetic data constructed with a varying number of samples n and features m and k equal sized tiles along the diagonal of the matrix (Supplementary Fig. 2). We only vary these variables because the runtime complexity of a single iteration of the MCMC (excluding sampling time) is $O(nmk)$. In each iteration, the likelihood of each sample vector is computed under each of the k existing clusters and the likelihood of each feature vector is computed under the signal and background models. Note that the value of k can change between iterations. To avoid fluctuating values of k significantly affecting runtime, the simulated data was constructed with a large difference between the signal (BetaBinomial(n=100, a=90,b=10)) and background (BetaBinomial(n=100, a=10,b=90)) distributions and runs of the algorithm were initialized with the correct number of clusters (using k-means). We ran the algorithm for 10 iterations on each dataset on a Intel(R) Xeon(R) Gold 6238R CPU @ 2.20GHz CPU machine with 512 GB of RAM and recorded the mean runtime per iteration.

To assess memory usage, we used the mprof utility from the python package memory_profiler. This tool samples the memory usage of the algorithm in 1 second intervals. We report the peak usage as the max memory (Mb) used across the sampled data points. Together, these results indicate that the runtime and memory increase with the number of features and samples. Increasing sample count increases runtime at a higher rate than increasing the number of features. Cluster count has a the largest effect on runtime and memory. For very large datasets, the runtime and memory usage appear to increase at different rates from the established trends (e.g. for the 2 cluster test, there was an increase in memory usage at 800 samples and 5000 LSVs but a decrease at 1000 samples and 5000 LSVs). This occurred because the CRP was opening singleton clusters which we hypothesize was due to increasing data complexity. However, these singleton clusters are still ultimately removed in the posterior summary process.

3 CHESSBOARD Pipeline and Features

Supplementary Note 3.1: Standard CHESSBOARD Pipeline for Real Data Analysis

This section details the default/standard settings and parameters used to run CHESSBOARD on our real datasets. We used MAJIQ to estimate read rates from STAR aligned BAM files. The median of the bootstrapped read rates for the most variable splice junction in each LSV was used as the representative junction in the data matrix. Note that CHESSBOARD supports non-MAJIQ processed input for any splicing quantification metric that can be interpreted as a ratio of junction inclusion or relative isoform proportions. We applied our filtering procedure to the data matrix using default parameters as described (Methods). We then ran CHESSBOARD on this data matrix with a k-means initialization (on Ψ) of k = 5 and conc = 1e-100. We used hyperpriors $\alpha_0 = \beta_0 = 0.5$ (Jeffery's prior modeling propensity for high or low junction inclusion) for background and $\alpha_1 = \beta_1 = 5$ (Beta prior modeling intermediate inclusion with

moderate variance) for the signal. For missing value priors, we estimated the values empirically on GTEX whole blood data using beta-binomial regression (Methods). The regularization parameter by default is 50. We ran the MCMC chain for 1000 iterations with a burn in of 200 and thinning step size of 2 but noted that the algorithm essentially converged at 20 iterations (model likelihood stopped changing after each iteration). We observed the same convergence using chains with alternate k-means initializations. This is because we are working with very high dimensional data. Although MCMC procedures are typically run for multiple chains where each chain is assessed for convergence using a stationary test diagnostic such as Heidelberger and Welch, we opt to use treat the optimization procedure using an expectation maximization approach with multiple start conditions given that model likelihood stop changing relatively quickly. This can be interpreted as an approximation for a variational inference approach where the MCMC is treated like a gradient based optimization.

Supplementary Note 3.2: Convergence Diagnostics

To enable alternative approaches for users to evaluate convergence of the MCMC samples to a stationary distribution, we implemented the Heidelberg-Welch test[7]. The hypotheses of the test are

- H_0: The chain is from the stationary distribution.
- H_1: The chain is not from the stationary distribution.

The input for this test is the log-posterior likelihood Markov chain obtained by running the CHESSBOARD algorithm. For a chain of length n, subchains are constructed by removing the first z samples of the chain in increments of a specified step size up to half the chain length ($n/2$). For each subchain, we compute the statistic vector $B_z(t)$ for $\{t|0 \leq t \leq n \wedge nt \in \mathcal{Z}^+\}$ where $\mathcal{Z}^+$ is the set of positive integers and θ_j is the jth element of the subchain.

$$
\begin{aligned}
T_k &= \sum_{j=1}^{k} \theta_j \\
\bar{\theta} &= \frac{\sum_{j=1}^{n-z} \theta_j}{n-z} \\
B_z(t) &= \frac{T_{\lceil (n-z)t \rceil} - \lceil (n-z)t \rceil \bar{\theta}}{\sqrt{(n-z)S(0)}}
\end{aligned} \tag{8}
$$

Here, $S(0)$ is a scalar defined as the spectral density of the latter half of the full chain at frequency zero. There are multiple approaches to computing $S(0)$ but we use an auto-regressive model approach by first fitting the following auto-regressive model with lag p to the chain. The degree (lag) of the model was selected using AIC.

$$
\theta_j = \sum_{i=1}^{p} \beta_i \theta_{j-p} + \epsilon \tag{9}
$$

Then we compute $S(0)$ using the Yule-Walker method.

$$
S(0) = \frac{Var(\epsilon)}{(1 - \sum_{i=1}^{p} \beta_i)^2} \tag{10}
$$

To compute a p-value for this test, we compute the test statistic

$$
\int_0^1 B_z(t)^2 dt \tag{11}
$$

We approximate the integral using a Reimann sum and compute the p-value using the CDF for the Cramer-Von-Mises Distribution. Please note that the null distribution indicates that the chain is stationary. Thus the first subchain that has a non-significant p-value is used since we cannot reject the null.

To further assess the robustness of CHESSBOARD's posterior distribution, we applied the algorithm to three synthetic datasets and evaluated convergence of the MCMC (Supplementary Fig. 3). In each dataset (samples = 30, LSVs = 30), LSVs were simulated from the same Beta-Binomial distribution to ensure that there was a consistent difference in variability between datasets. The distribution and tile structure used in each dataset is shown in Supplementary Fig. 3. The left most dataset has the highest variability while the right most has the lowest. We ran CHESSBOARD on each datset for 2000 MCMC iterations with $\alpha = 0.1$ and a k-means initialization of $k = 5$. In the highest variance dataset, 5 clusters were found. Although this does not match the ground truth cluster number from the the simulation, the result is reasonable as shown by the pairwise clustering probability plot. The samples from both clusters are clearly separated as there is 0 probability of them clustering together. However, within each of the original clusters, there appear to be 2 subclusters. One is a high confidence group (higher probability of samples clustering together/darker color in the heatmap) and the other is a low confidence group (low probaiblty of samples clustering together/lighter color in the heatmap). The final cluster is an outlier group with samples that are a poor fit for all of the other clusters. The marginal probabilities of each cell in the matrix belonging to the signal distribution also shows high variability. Note that the signal-background distribution designation does not matter here since the clusters are equal in size. The tile structures still are correctly identified (just at a finer resolution). The Heildelberg-Welch diagnostic indicates that the log-posterior of the model has converged to the stationary distribution after 229 iterations (step size = 1, p = 0.695). As the variability of the datasets increase, the variance of the posterior begins to decrease. The second dataset converges at 114 iterations (step size = 1, p = 0.114). The third dataset converges almost instantly (step size = 1, p = 0.320). This is due to extremely low data variability which results in a low variance posterior with high confidence clusters. It should be noted that this dataset is representative of our realistic simulated data (Supplementary Note 2.3) and our real

data (Supplementary Note 1.1). Consequently, the algorithm converges very quickly to a stable solution on these datasets. Furthermore, these datasets are very high dimensional in comparision to the datasets used in this analysis which makes it much harder for the algorithm to explore the full posterior. For such data, it is recommended to use the convergence evaluation criteria described in Supplementary Note 3.1.

Supplementary Note 3.3: Using CHESSBOARD as a Predictive Model

To use CHESSBOARD as a predictive model, we want to assess the probability of a sample being assigned to one of the clusters. This is given by

$$P(c_i = z|x_i) \propto [r_z P(x_i|\alpha_1, \beta_1) + (1 - r_z)P(x_i|\alpha_0, \beta_0)]P(c_i = z) \quad (12)$$

CHESSBOARD learns the parameter values from a training dataset by using the parameters from the sample that minimizes MSE to the posterior mean. We can then predict the likelihood of a sample given all the learned parameters using a mixture of Beta Binomial likelihoods. We used this approach to predict clustering assignments for the samples in the Penn HTSC dataset. First, we ran the algorithm on the beatAML datasets to learn all of the above parameters. Since there were 2 clusters, $z \in [1, 2]$. Then we assigned 2 likelihoods to each sample in the Penn HTSC dataset. One for $z = 1$ and one for $z = 2$. Finally, we made a hard clustering assignment by placing the sample in the cluster for which it had the higher likelihood.

Supplementary Note 3.4: Statistical Testing in Regulation Analysis with ENCODE Data

In this section, we detail the statistical tests used to generate Fig. 3f. To determine if a RBP/SF is differentially expressed between the signal and background clusters, we use DeSeq2 on Salmon transcript expression quantifications. If a gene has multiple quantified transcripts, we consider the gene differentially expressed if at least one transcript is DE. A transcript is DE if its log2FC

> 1 and its bonferroni corrected p-value is < 0.05. To determine if a RBP/SF is differentially spliced, we use MAJIQ Het on MAJIQ quantifications. If a gene has multiple LSVs, we consider the gene differentially spliced if at least one junction is DS. A LSV is DS if its median $\Delta\Psi$ is > 0.2 and bonferroni corrected Wilcoxon p-value is < 0.05 for a junction. To determine which splice junctions were regulated by each RBP/SF, we performed a differential splicing analysis using MAJIQ between RBP/SF knockdown samples and controls. For each RBP/SF, there were 2 replicates for knockdowns and 2 for controls. The RBP/SF knockdown experiments in the ENCODE dataset were generated in 32 batches in K562 cell lines. We used MOCCASIN to correct for batch effects. The controls were considered all together as one group. Any junction with posterior probability of $\Delta\Psi > 0.2$ was considered differentially spliced and thus regulated by the RBP/SF. To determine which junctions had CLIP binding of the RBP/SF, we first identified binding regions by assessing whether the Irreproducible Discovery Rate (`https://www.encodeproject.org/data-standards/terms/#concordance`) p-value was < 0.05 for CLIP peaks generated from 2 replicates. Then we checked whether the region bounded by the RBP overlapped with a 250 bp window flanking each side of the junction. A window that overlaps with at least one binding region indicated that the junction had CLIP binding. To compute p-values for enrichment of overlap, we used a 1 sided fisher's exact test for enrichment on the following 2 x 2 table. The null hypothesis for the 1 sided test is that the odds ratio of junctions in tile to junctions not in tile is greater than 1.

	Junction in Tile	Junction not in Tile
Regulated/Binding/Both	Junction in Tile & Regulated/Binding/Both	Junction not in Tile & Regulated/Binding/Both
∼(Regulated/Binding/Both)	Junction in Tile & ∼(Regulated/Binding/Both)	Junction not in Tile & ∼(Regulated/Binding/Both)

Regulated indicates the junction was in a LSV that was determined to be regulated in the ENCODE analysis. Binding indicates CLIP binding of the RBP/SF was observed in the ENCODE

data near the junction. Both is the intersection of regulated and binding.

Supplementary Note 3.5: CHESSBOARD can Rank Tile's Splicing Events for Downstream Analysis

In many genomic analysis tasks such as differential splicing or gene expression researchers are interested in a ranked list of entities (e.g. mutations, genes), which they then test for enrichment of some biological signal (e.g. pathways). Ranking is also desirable since the tile structure is clearly an approximation of the underlying biological signals. Specifically, some LSV may exhibit a strong pattern that closely matches the patients subgroups thus "driving" the tile formation while others can be considered as "passengers" with a much less clear pattern. However, standard differential splicing analysis can not be applied in this setting as it is based solely on observed Ψ and ignores the missingness signal discussed above. Thus, to address the need for splicing changes ranking we developed a LSV ranking procedure that takes advantage of CHESSBOARD's probabilistic framework. The ranking score is computed as

$$Rank(LSV_j) = \sum_{i}^{N}[log(P(x_{ij}|\alpha_{j1}, \beta_{j1}, c_i, r_{c_i})) - log(P(x_{ij}|\alpha_{j1}, \beta_{j1}, c_i, r*_{c_i}))] \quad (13)$$

The first term represents the likelihood of the LSV under the learned tile model while the second term represents the likelihood under an inverted model. Recall that the likelihood under the tile model is computed as the mixture of a signal and background Beta distribution where subscript 1 indicates signal and subscript 0 indicates background.

$$P(x_{ij}|\alpha_{j1}, \beta_{j1}, c_i, r_{c_i}) = r_{c_i}P(x_{ij}|\alpha_{j1}, \beta_{j1}) + (1 - r_{c_i})P(x_{ij}|\alpha_{j0}, \beta_{j0}) \quad (14)$$

Under the inverted model, we compute the likelihood in the same way except $r*_{c_i}$ is defined as $r*_{c_i} = 1 - r_{ci}$. Intuitively, the ranking score is the total likelihood a LSV "gained" from the learned model compared to the alternative. If the the signal and background distributions are similar (i.e. KLD is low), then the data has similar likelihood under both models. This

indicates that the LSV does not strongly drive tile structure because a sample would have approximately equal probability of being assigned to signal or background if classified using this feature alone. Conversely, LSVs with a high score gain substantial likelihood from the learned tile configuration and strongly contribute to the tile structure. When applied to the tiles derived from the AML associated genes, we find a distinct exponential shape of the score distribution. This result indicates that a few "driver genes" define the tile shape while the majority of LSV features contribute much less to the structure (Supplementary Fig. 5a). We then confirmed that the score is directly correlated with the amount of separation between modalities. We observed that the highest ranking LSV has large separation while LSVs with scores in 75th, 50th and 25th percentile show decreasing separation (Supplementary Fig. 5b). The modalities in the lowest scoring LSV were almost completely overlapping. Finally, we assessed the rankings of the notable LSVs we analyzed in the previous section. *FLT3* LSV1 and LSV2 rank near the 50th percentile while the two *EZH2* LSVs ranked lower. However, we note that *EZH2* was prioritized due to its missingness pointing to the importance of our missing value model. The top ranking LSV was *U2AF1* which is expected to regulate a substantial number of the events in the tile given the regulatory analysis described above.

4 beatAML Analysis

Supplementary Note 4.1: Recursive Clustering and Termination

The CHESSBOARD framework naturally enables recursive clustering through its tile based clustering approach and probabilistic framework. The recursive clustering algorithm is presented below.

Algorithm 1: Recursive Clustering

$M_0 = CHESSBOARD(X_{F_0})$;
$\bar{X}_{F_0} = Shuffle(X_{F_0})$;
$M_0* = CHESSBOARD(\bar{X}_{F_0})$;
$LR_0 = median(LL(X_{F_0}, M_0) - LL(\bar{X}_{F_0}, M_0*))$;
while $|LR_n - LR_{n-1}| < T$ **do**
  $F_n = \{LSV_j | \forall j\ s.t. \sum_c r_{jc} = 0\}$;
  $M_n = CHESSBOARD(X_{F_n})$;
  $\bar{X}_{F_n} = Shuffle(X_{F_n})$;
  $M_n* = CHESSBOARD(\bar{X}_{F_n})$;
  $LR_n = median(LL(X_{F_n}, M_n) - LL(\bar{X}_{F_n}, M_n*))$;
end

Let X represent a data matrix with rows representing all LSVs in the transcriptome and columns representing all patient samples in the dataset. Define F_0 as the feature set of the initial input matrix (i.e. all features that pass the pre-filtering pipeline). We apply CHESSBOARD to the matrix X_{F_0} to obtain posterior model M_0. M_0 represents all latent posterior random variables learned from the data. We then generate a null matrix with the same feature set denoted as $\bar{X}_{F_0}$ by shuffling the rows of X_{F_0}. The shuffling procedure involves independently and randomly permuting each row of the input matrix to break tile structure. Next, we obtain the posterior model M_0* by running the algorithm on $\bar{X}_{F_0}$. We then use both posterior models to evaluate the log likelihood ratio LR_0 of each LSV in X_{F_0} under model M_0 to $\bar{X}_{F_0}$ under model M_0*. To conduct the first recursive step, define feature set containing LSVs not assigned to a tile F_n as $\{LSV_j | \forall j s.t. \sum_c r_{jc} = 0\}$. We then apply CHESSBOARD to X_{F_n} and null matrix $\bar{X}_{F_n}$ to obtain model M_n and M_n* respectively as in the base case. This procedure is repeated until the termination criteria is met. We terminate the algorithm once the median LR stops changing from the previous iteration. We use the median LR of the LSV LR distributions so the likelihoods are comparable between iterations. Using the likelihood of the entire matrix for example would not be comparable since the cardinality of the feature sets decrease after each iteration. Non-changing can be defined as either the difference between medians being

below some threshold T or a test can be used to reject the null hypothesis that the likelihood means are unequal.

We also present an augmented version of the recursive algorithm. In our applications, we did not notice any major differences between the two versions of the algorithm and opted to not use the augmented algorithm due to run-time considerations. Under this approach, feature sets F are not confined to only LSVs in the input data matrix but include all LSVs in the transcriptome which are correlated to the unique structures that contain the LSVs in F. Specifically, for each unique binary vector r_j, divide the samples i into groups such that $r_{jc_i} = 0$ and $r_{jc_i} = 1$. Remove any LSVs from X that are differentially spliced between the groups. Once all LSVs correlated to each unique r_j have been removed, proceed to the next recursive step. Next, when generating the null model, instead of generating a single model, we can generate a bootstrapped empirical distribution over models through multiple permutations of X. Formally, we have $\bar{X}^1, \bar{X}^2 \ldots \bar{X}^B$ where B is the number of bootstrapped samples. Then we can evaluate whether the likelihood X_F is significant in the context of the distribution of $\bar{X}^{1,2,\ldots,B}$ and terminate the algorithm if the likelihood of X_F is an outlier.

Supplementary Note 4.2: Multiple Testing Correction with Missing Data

To correct for the family wise error rate in multiple testing, we use a min-P procedure. Let $C = \{c_i | \forall i \in [N]\}$ be the set of cluster labels for all N samples. For each mutation m out of M total mutations, we compute an observed p-value p_m using a two-sided fisher's exact test on the following 2 x 2 matrix.

	$c_i = 0$	$c_i = 1$
Mutation+	Mutation+ & $c_i = 0$	Mutation+ & $c_i = 1$
Mutation-	Mutation- & $c_i = 0$	Mutation- & $c_i = 1$

Missing mutation annotations are ignored. We then generate a bootstrapped null p-value distribution. For each of B bootstrapped samples, we randomly permute C such that each c_i

takes on the value of a random $c_i \in C$ without replacement. Then given these new clustering assignments, we compute fisher's exact test again for each mutation to obtain $p*^b_m$. For each bootstrapped sample, we record the minimum p-value across all mutations $minP_b = min(p*^b_1, p*^b_2, \ldots, p*^b_M)$. To compute the corrected p-value for a mutation m, we count the number of times the observed p-value $\sum_b I(p_m > minP_b)$ and divide by B.

5 Drug Response Analysis

Supplementary Note 5.1: Drug Response Correlation

Drug response in the BeatAML dataset was quantified using two measurements: IC_{50} and AUC. IC_{50} is the concentration (μM in the beatAML study) at which a drug inhibits a target biological process by 50%. In the beatAML study, inhibition was measured as the normalized cell viability which is a quantity derived from the optical density of surviving tumor cells in a plate after treatment with the drug. The IC_{50} value for each drug was obtained (by the beatAML study) by fitting a sigmoid curve to 7 data points defined as the tuple (concentration, inhibition) and selecting the concentration at which inhibition is 50% of its maximum value. Each of the 7 concentrations represents a 3 fold dilution starting at $10\mu M$ and ending at $0.0137\mu M$. When we compare IC_{50} changes in this study, we use a log3 transform since each unit change of IC_{50} in log3 space is 1 fold change unit given the 3 fold dilution. AUC is the area under the IC_{50} curve which is an unbounded positive quantity and a larger value indicates poor drug response. We chose to use AUC over raw IC_{50} because concentration ranges used in the beatAML experiments limited fitting of sensitivity curves. However, we note that the median AUC and IC_{50} changes between the groups in beatAML were highly correlated (r = 0.727) indicating that quantitative comparisons in AUC space translated to IC_{50} space (Supplementary Fig. 6a). Interestingly, most outliers in this figure (i.e cases when there is low agreement between IC_50 and AUC changes) had median IC_{50} values of 10 in both clusters ($\Delta IC_{50} = 0$) and were likely

the most affected by the concentration range. This can occur when there is no sigmoid curve but rather a horizontal line due to the fact no concentration in the chosen range produced any noticeable effect. In this case, relative AUC quantities can still be compared but IC_{50} cannot. A change in AUC quantity should thus be used to assess whether there is difference in response between groups. However, the difference itself does not have a clear interpretation. Instead, given a significant difference in AUC, one can then look for a large fold change in IC_{50} to determine if the change is biological meaningful. The patient groups differ most significantly by their sensitivity to JQ1 (p = 7.21e-05, Δmedian(AUC) = 25.76) (Supplementary Data 4) based on a Kruskal-Wallis test. The tile group had a higher median IC_{50} compared to the background group corresponding to a Log3 fold change of 0.773 indicating a higher drug sensitivity in the background (Supplementary Data 4). The drugs Tramatenib (p = 5.77e-03, median(ΔAUC) = 42.50, Log3FC = 2.65) and Venetoclax (p = 1.56e-02, Δmedian(AUC) = 38.08, Log3FC = 2.34) were the two highest ranking drugs in terms of AUC and IC_{50} change and are common drugs administered to AML patients. In contrast, drugs with poor correlation are associated with other conditions. For example, Vemurafenib (p = 0.831, Δmedian(AUC) = 1.39) is a Melanoma drug and Lovastatin (p = 0.962, Δmedian(AUC) = 8.80) is intended to reduce cholesterol levels. To assess potential functional significance, we first looked for differential splicing of specific gene targets of these drugs. Specifically, we observed differential splicing at multiple junctions in *BRAF* (JQ1 target), *MAP2K1* (Tramateib target) and *BCL2* (Venetoclax target).

Supplementary Note 5.2: Variance Explained

$$Var(IC_{50}) = E[Var(IC_{50}|\textit{FLT3} - ITD^{+})] + Var[E(IC_{50}|\textit{FLT3} - ITD^{+})]$$
$$VarianceExplained(\textit{FLT3} - ITD^{+}) = \frac{Var[E(IC_{50}|\textit{FLT3} - ITD^{+})]}{Var(IC_{50})} \tag{15}$$

Supplementary Note 5.3: Decision Tree Permutation Test

To compute whether AUC gained from including splicing profiles in the decision tree was significant, we used a permutation test. To construct the decision tree for a specific drug and mutation pair, we first split samples based on $M_i = +$ or $M_i = -$ and then conduct a second split based on splicing profiles (i.e. cluster identity). We use variance explained by the decision tree as the test statistic. The null hypothesis for this test is that combining the mutation and splicing classification does not improve drug response prediction compared to using mutation status alone. The alternate hypothesis is that the combined classification improves drug response prediction compared to using mutation status alone. For the observed test statistic, we use the variance explained of our decision tree in Fig. 5c. We then compute 1000 bootstrapped trees where we randomly permute clustering assignments into equal sized groups for the second split. To compute a p-value, we use $I(VarExp_{boot} > VarExp_{obs})/1000$.

6 Model Details

Supplementary Note 6.1: Variable Table

Variable Definitions		
Variable	Description	Distribution
n	The total number of samples or number of matrix columns. Samples indices are denote as $i \in \{1, 2, \ldots, n\}$	NA
m	The total number of LSVs or number of matrix rows. LSV indices are denote as $j \in \{1, 2, \ldots, m\}$	NA
x_{ij}	The number of reads mapped to the representative splice junction of LSV j in sample i.	Binomial
η_{ij}	The total number of reads mapped to LSV j in sample i.	NA
ω_{ij}	An indicator variable which denotes whether LSV j in sample i is missing/unquantifiable. $\omega_{ij} = \begin{cases} 1 & \text{if observation is missing} \\ 0 & \text{otherwise} \end{cases}$	Bernoulli
c_i	Denotes assignment of sample i to cluster k if $c_i = k$.	CRP
r_{jk}	Denote assignment of LSV j for all samples such that $c_i = k$ to either the background ($r_{jk} = 0$) or signal ($r_{jk} = 1$) distributions.	Bernoulli
Ψ_{ij}	Percent splice in of LSV j in sample i	Beta
θ_{js}	Missingness rate of LSV j for the signal distribution $s = 1$ or background distribution $s = 0$	Beta
μ_{js}	Mean of Ψ of LSV j for the signal distribution $s = 1$ or background distribution $s = 0$	Beta
κ_{js}	Concentration/inverse variance of Ψ of LSV j for the signal distribution $s = 1$ or background distribution $s = 0$	Prior
α_{js}, β_{js}	The priors for the Beta distribution modeling μ_{js}	Hyperprior
a_{js}, b_{js}	The priors for the Beta distribution modeling missingness rate θ_{js}	Prior
$\sum_k r_{jk}$	Regularization term.	Exponential
λ	Regularization hyperparmeter.	Hyperparameter
α_o	CRP concentration parameter	Hyperparameter

7 Survival Analysis

Supplementary Note 7.1: Survival Analaysis

We performed a survival analysis on the beatAML samples. Patients were divided into two groups based on whether they were assigned the signal or background cluster (Fig 3a). Out of the 217 samples in the signal cluster, 132 had survival data (60.83%). Out of 260 samples in the background cluster, 184 had had survival data (70.77%). There were no censored observation in which patients dropped out of the study for reasons other than the event of interest (death). We generated the survival curves for the background and signal groups denoted as $S_0(t)$ and $S_1(t)$ respectively for all available time points t using the Kaplan-Meier method (Supplementary Fig. 7). To assess whether the survival distributions were significantly different, we used the log rank test which is the standard in the field. The hypotheses of the test are

- $H_0 : S_0 = S_1$
- $H_0 : S_0 \neq S_1$

The log-rank p-value was 0.6265. Although this is not significant, it is likely due to the fact that the log rank test is under-powered to detect differences in non-diverging survival distributions[8]. The log rank test is a multi group extension of a chi-squared test which assesses whether the observed cumulative deaths (after accounting for censored data) in each group different significantly from the expected cumulative deaths in the dataset (i.e. groups are combined). Thus the test is under-powered when the survival curves converge resulting in similar cumulative death totals. In such scenarios, any divergence or omnibus test would be more appropriate. Since we do not have censored data, we can also assess significance using a 2 sample Kolmogorov-Smirnov test. The KS test statistic is $D = 0.158$ and the KS p-value is 0.054. The test statistic can be interpreted as the largest difference in survival probability between the

groups which is 15.8%. Specifically, The survival of the first seven months after diagnosis is similar and the ultimate survival after several years is also the same. Only in between these two time points some difference could be observed. While this represents a statistically significant difference in survival rate and we report it here for completeness, the biological significance of this difference is unclear and also difficult to explain.

Supplementary Figures

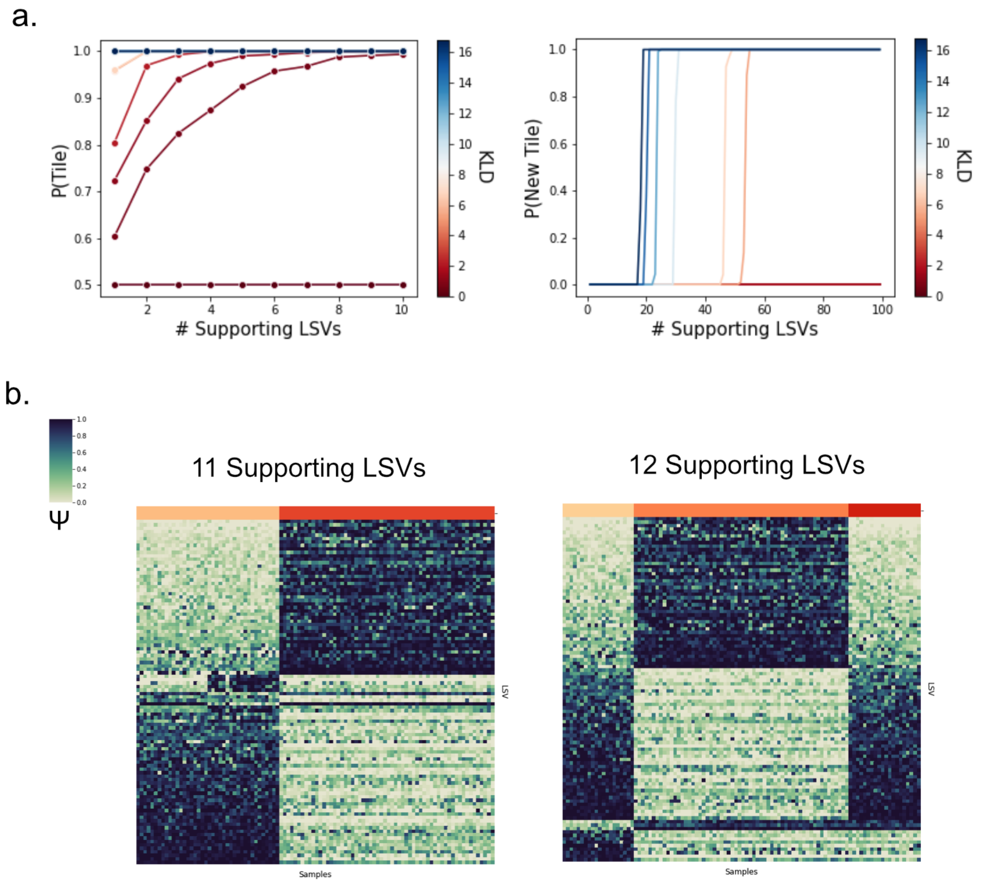

Supplementary Fig. 1: Additional Model Evaluation.

(A) Probability of cluster discovery based on evidence in data. LEFT: Probability of the CHESSBOARD model assigning a sample to the tile/signal distribution of equal size (i.e. k = 2 initialization) as a function of increasing KL divergence (red low, blue high) w.r.t to a background Beta distribution of Beta(10,1). and number of supporting LSVs in the tile. RIGHT: Probability of assignment when discovering new tiles with the concentration parameter set using an effective dimensionality (Supplementary Note 2.2) of 50. **(B)** Number of supporting LSVs required to identify tile in realistic synthetic data. Left heatmap shows the clustering result on data with 3 tiles where one of the tiles only has 11 supporting LSVs. The algorithm is however only able to find the 2 main tiles. Right heatmaps shows the clustering result when the 3rd tile has 12 supporting LSVs. The algorithm is able to successfully find all 3 tiles.

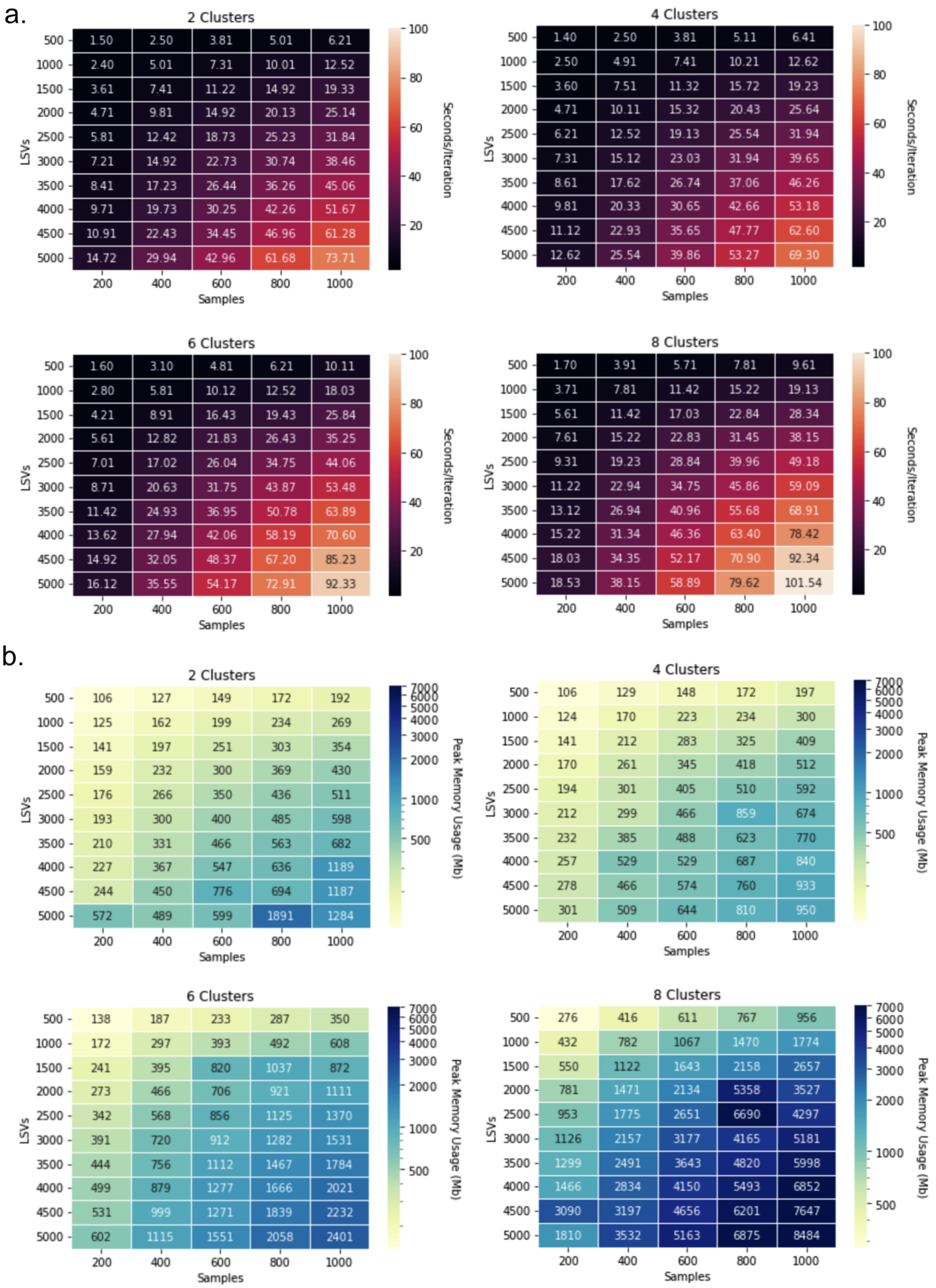

Supplementary Fig. 2: Runtime and Memory Evaluation.

(A) Heatmaps showing the runtime in seconds per MCMC iteration of CHESSBOARD when run on synthetic data with the specified number of samples, LSVs and clusters. **(B)** Heatmaps showing peak memory usage in Mb of CHESSBOARD when run on synthetic data with the specified number of samples, LSVs and clusters.

Signal Distribution	BetaBinomial(n=10,a=5,b=15)	BetaBinomial(n=20,a=10,b=30)	BetaBinomial(n=50,a=20,b=60)
Simulated Data Ψ 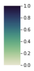	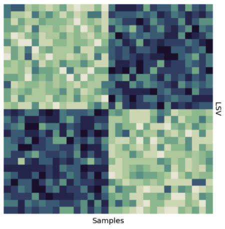	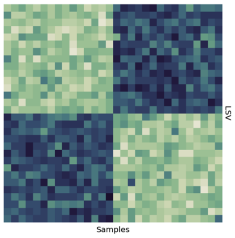	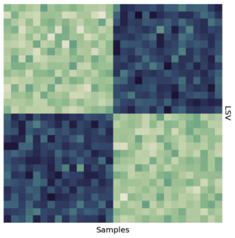
Pairwise Clustering Probabilities P 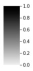	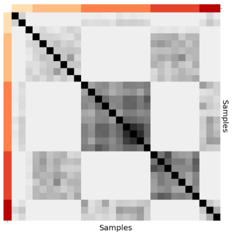	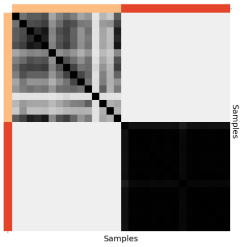	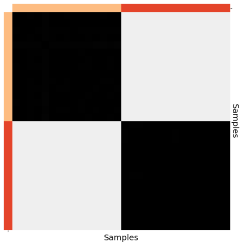
Marginal Signal Probabilities P 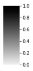	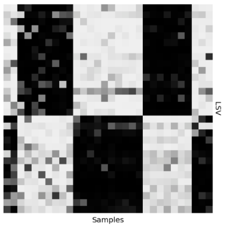	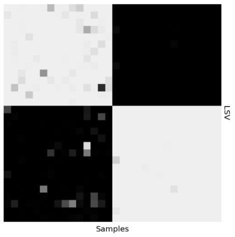	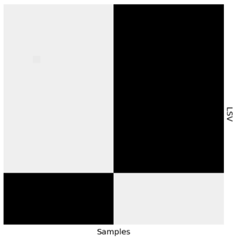
Clustering Ψ 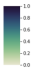	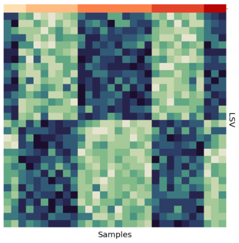	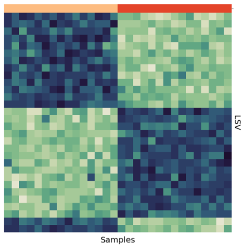	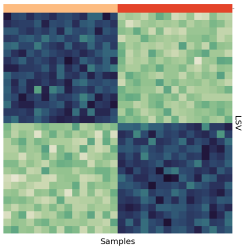
Log-Posterior Traceplots	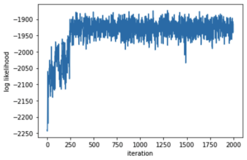	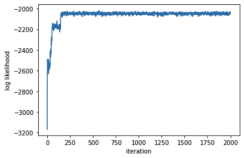	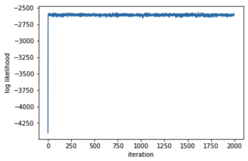
Heidelberger-Welch Diagnostic	Stationary Distribution: [229,2000] p-value: 0.695	Stationary Distribution: [114,2000] p-value: 0.114	Stationary Distribution: [1,2000] p-value: 0.320

Supplementary Fig. 3: MCMC Convergence Evaluation.

CHESSBOARD applied to various synthetic datasets with different levels of variability. The first row of the table shows the signal distribution for each simulated dataset. The background distribution reverses α and β. The variance of the datasets increases from left to right. High variability results in the algorithm finidng more clusters while low variance results in a very stable solution. The traceplots and Heidelberger-Welch diagnostics show that convergence to stationary distribution occurs much faster in the low variance dataset compared to the high variance dataset.

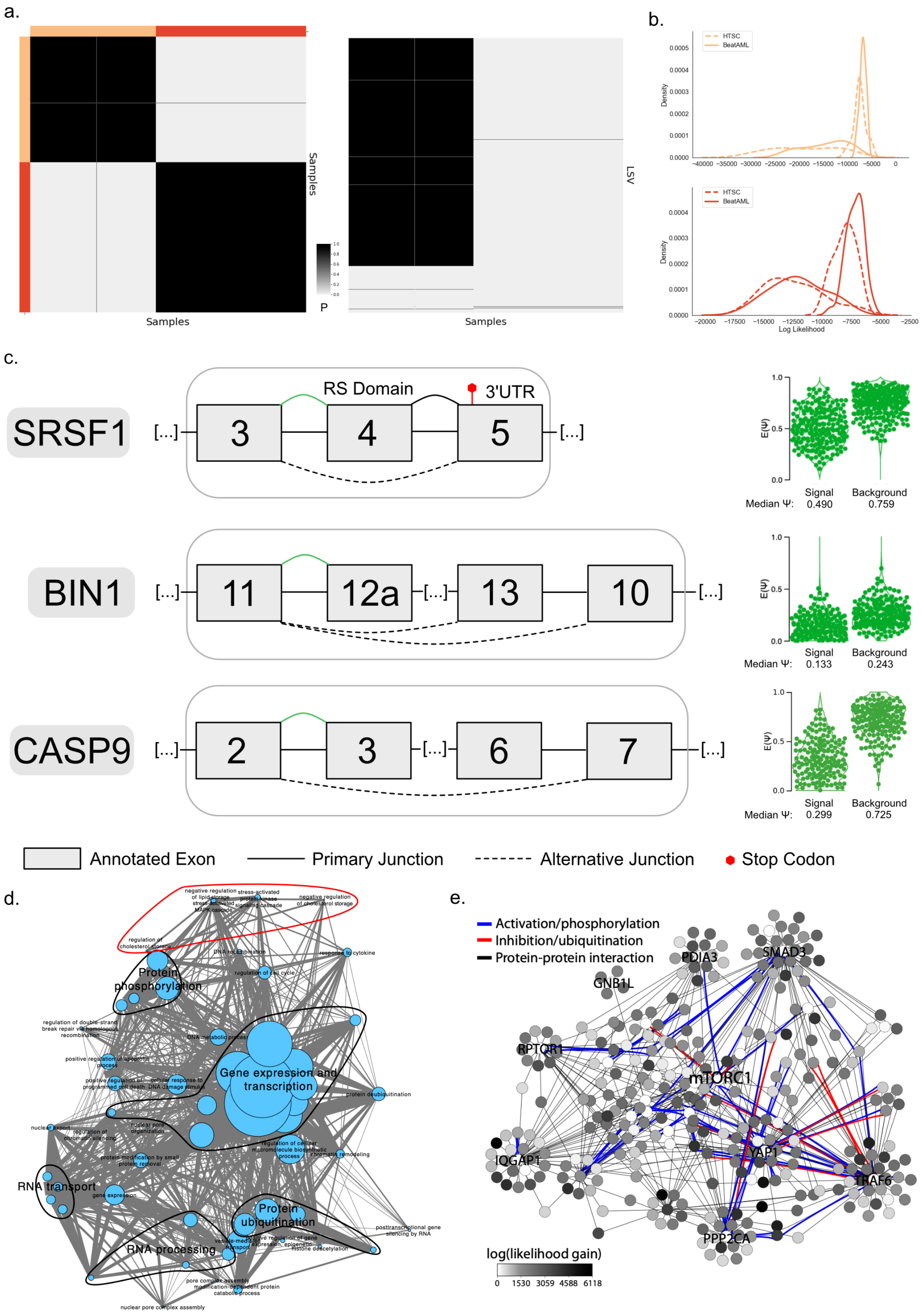
a.
Samples
P
Samples
LSV
b.
HTSC
BeatAML
Density
Log Likelihood
c.
SRSF1
RS Domain
3'UTR
[...]
3
4
5
BIN1
11
12a
13
10
CASP9
2
3
6
7
E(Ψ)
Signal
Background
Median Ψ:
0.490
0.759
0.133
0.243
0.299
0.725
Annotated Exon
Primary Junction
Alternative Junction
Stop Codon
d.
Protein phosphorylation
Gene expression and transcription
RNA transport
RNA processing
Protein ubiquitination
nuclear pore complex assembly
e.
Activation/phosphorylation
Inhibition/ubiquitination
Protein-protein interaction
PDIA3
SMAD3
GNB1L
RPTOR1
mTORC1
IQGAP1
YAP1
TRAF6
PPP2CA
log(likelihood gain)
0
1530
3059
4588
6118

Supplementary Fig. 4: Additional beatAML Data Analysis.

(A) Left: Pairwise clustering probabilities representing the frequency that a pair of samples were assigned to the same cluster in a MCMC iteration. Right: Marginal probabilities representing the frequency that a matrix entry was assigned to the signal/tile distribution in a MCMC iteration. Both statistics indicate highly stable tiles. **(B)** Sample log likelihood distributions under the signal model (left) and background model (right). Solid curves represent the held out beatAML samples while dotted curves represent the Penn HTSC dataset. The curve with higher average likelihood in the signal plot corresponds to samples that were assigned to the signal cluster while the curve with lower average likelihood captures the samples that were assigned to the background cluster. The curve with higher average likelihood in the background plot corresponds to samples that were assigned to the background cluster while the curve with lower average likelihood captures the samples that were assigned to the signal cluster. **(C)** Splicing in *SRSF1* has higher inclusion of exon 4 ($\Delta\Psi$ = 0.269) in the background group which contains the RS domain. Exclusion results in a transcript that encodes a truncated protein and is targeted for NMD. *BIN1* and *CASP9* are targets of *SRSF1* mediated splicing regulation. There is increased inclusion of exon 12a ($\Delta\Psi$ = 0.110) in the background and increased inclusion of exons 3-6 in *CASP9* ($\Delta\Psi$ = 0.426). Both inclusion events are associated with *SRSF1* overexpression. **(D)** Enrichment map showing GO terms related to biological processes that have at least one gene in common. Each node is a GO term; the thickness of edges corresponds to the number of genes in common between two nodes. GO terms related to stress-related cellular responses (regulation of cholesterol/lipid storage and MAPK-signaling) are highlighted in the red set. **(E)** mTORC1 network: Network of genes with LSVs that are part of the tile showing direct interaction with mTORC1 or a direct mTORC1 regulator through activation, phosphorylation, inhibition or ubiquitination activities or protein-protein interactions. Only interactions with experimental evidence, as curated in the IPA software, are shown.

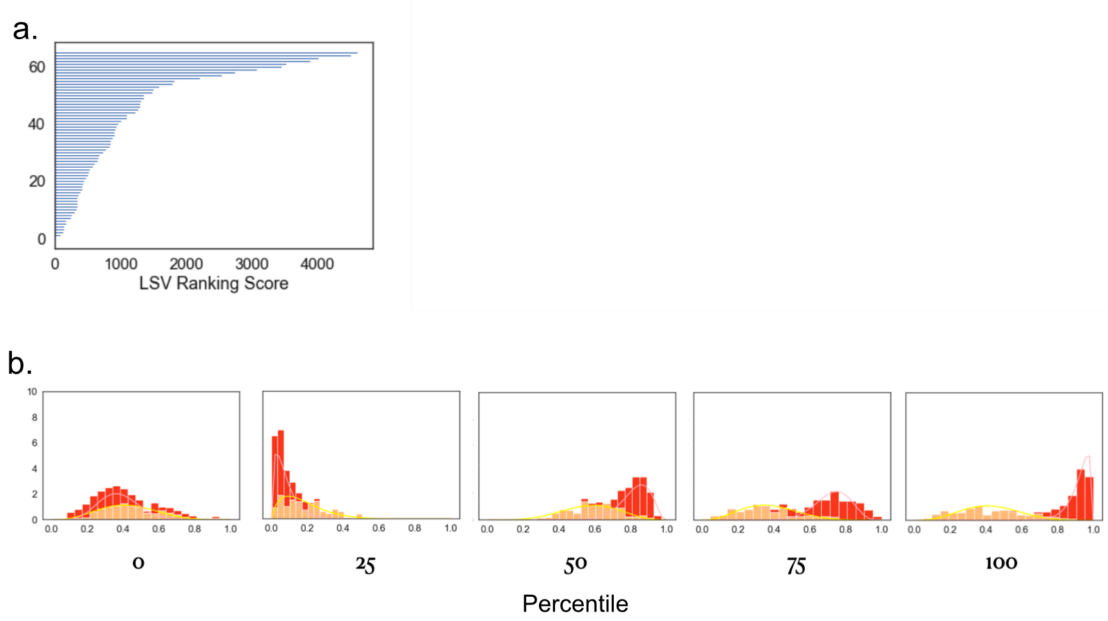

Supplementary Fig. 5: Gene Ranking Analysis.

(A) Barplot showing the ranking scores for each LSV in the tile discovered in the beatAML 70 gene data matrix. **(B)** Histograms showing the Ψ distributions (x-axis) for 5 different LSVs that rank at the 0th, 25th, 50th, 75th and 100th percentiles. The bars are colored based on the cluster data points are assigned to. Bars with multiple colors show the relative proportion of samples assigned to each cluster. Curves represent the Beta posterior distributions fitted by CHESSBOARD.

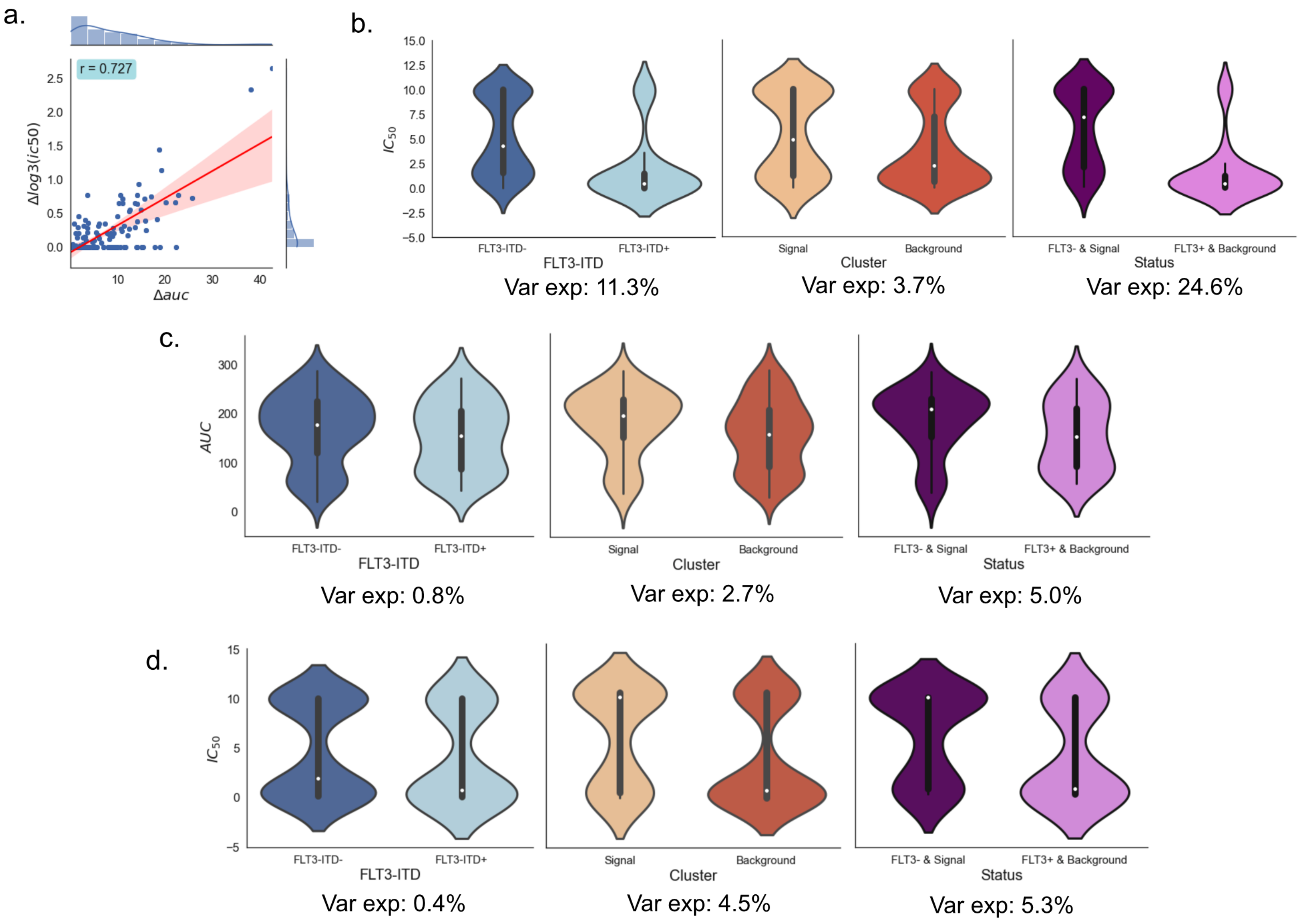

Supplementary Fig. 6: Additional Drug Response Analysis.

(A) Pearson correlation between $\Delta median(AUC)$ and $\Delta log3FC(IC_{50})$ between the 2 clusters discovered by applying CHESSBOARD to the beatAML dataset using only 70 AML related genes. The red line represents the slope of the fitted linear regression and the band around the line represents the 95% confidence interval of the regression coefficient. **(B)** Violin Plots show the distribution of IC_{50} for patients' response to Sorafenib when split according to the groups indicated on the x-axis. When using the splicing clusters as a predictive variable,

the variance explained is only 3.8% while this increases to 11.3% with *FLT3*-ITD status. When combining both sources of information using the decision tree in Fig. 5c, the variance explained increases to 24.6%. The bars at the top of Fig. 5d indicate the total number of samples that fall into each category. (C) Violin Plots show the distribution of AUC for patients' response to Venetoclax when split according to the groups on the x-axis. There are 107 mutation positive and 366 mutation negative samples (left violin plot), 212 signal and 262 background samples (middle violin plot), and 168 mutation negative + signal and mutation positive + background samples (right violin plot). (D) The same plots as (C) except for IC_{50}. The group sample sizes are the same as (C).

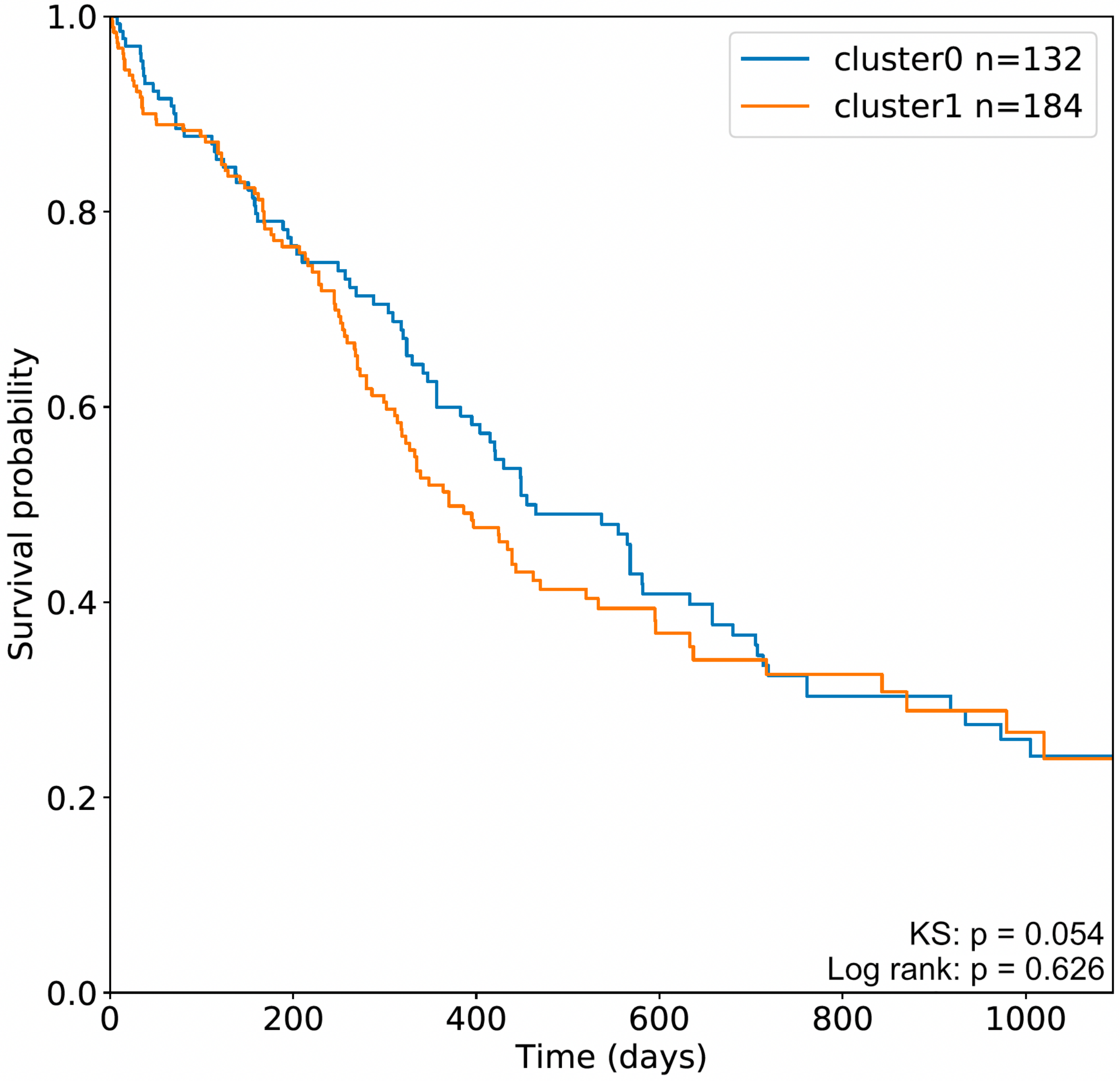

Supplementary Fig. 7: Survival Analysis.

Kaplan-Meier plot of survival probability between patients in the 2 clusters found in the genome wide analysis. The p-values were computed using a two-sided Kolmogorov-Smirnov (KS) test (H_1: the curves are not identical) and two-sided log rank test (H_1: the difference in survival probabilities $\neq 0$).

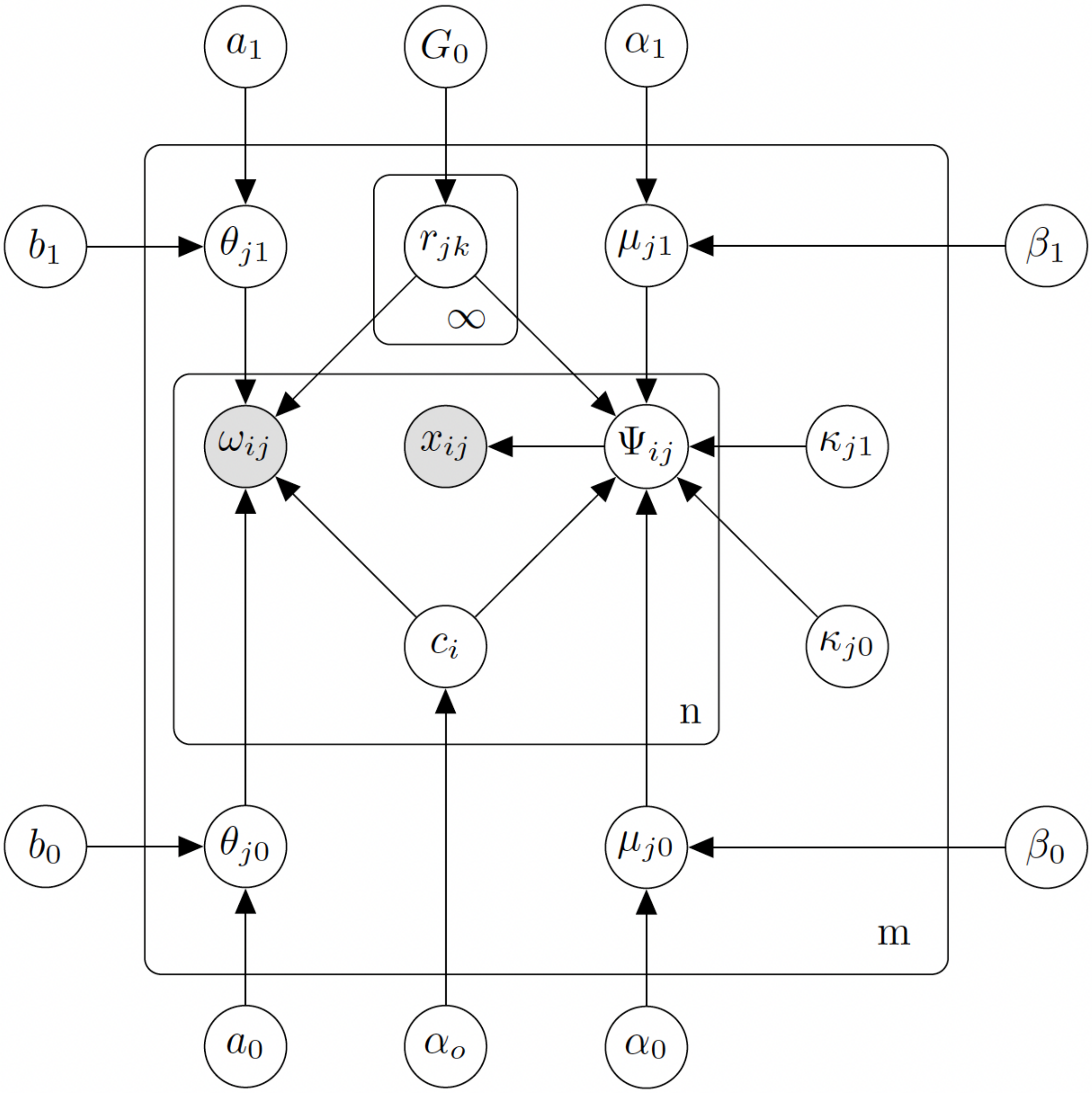

Supplementary Fig. 8: Plate Diagram.

A plate model showing the relationship between latent variables in a model. An arrow indicates the child node is dependent on the parent. Observed variables are shown in grey. Latent variables are white. See variable table in Supplementary Note 6.1.

Supplementary Data

The Supplementary Data include the human readable output of CHESSBOARD on each of our datasets. These are formatted as excel files where each tile k is defined by the contents under 3 tabs: Sample k, Cluste k and Background k. Sample denotes the sample IDs in the tile. Cluster defines the LSVs that belong to the signal group in the tile. Background defines the LSVs the belong to the background group in the tile. There are 2 additional tables in each file: Consensus Background and Probability Missing Signal. Consensus Background defines all LSVs that don't belong to a signal in any tile. Probability Missing Signal defines the p-values that the signal group for a given LSV is enriched for missing values based on fisher's exact test. All files can be found in the Zenodo repository at https://zenodo.org/record/7245323#.Y1apPFLMKQc.

Supplementary Data 1: beatAML

Format defined above for the beatAML dataset.

Supplementary Data 2: beatAML Recursive Step 1

Format defined above for the first recursive step applied to the beatAML dataset.

Supplementary Data 3: beatAML AML Genes

Format defined above for the beatAML dataset using only AML related genes.

Supplementary Data 4: Drug p-values

Kruskal-Wallis p-values for differential drug response (measured as AUC) between the clusters discovered in the beatAML dataset using AML genes.

Supplementary Data 5: TARGET AML

Format defined above for the joined dataset of beatAML and TARGET pediatric AML datatsets.

Supplementary Data 6: TARGET B-ALL

Format defined above for the TARGET B-ALL dataset.

Supplementary References

[1] Tyner, J. W. *et al.* Functional genomic landscape of acute myeloid leukaemia. *Nature* **562**, 526–531 (2018).

[2] Rivera, O. D. *et al.* Alternative splicing redefines landscape of commonly mutated genes in acute myeloid leukemia. *Proceedings of the National Academy of Sciences* **118** (2021).

[3] Van Nostrand, E. L. *et al.* A large-scale binding and functional map of human RNA-binding proteins. *Nature* **583**, 711–719 (2020).

[4] Slaff, B. *et al.* MOCCASIN: A method for correcting for known and unknown confounders in RNA splicing analysis. *Nature Communications* **12**, 1–9 (2021).

[5] Shen, S. *et al.* rMATS: robust and flexible detection of differential alternative splicing from replicate RNA-Seq data. *Proceedings of the National Academy of Sciences* **111**, E5593–E5601 (2014).

[6] Vaquero-Garcia, J. *et al.* A new view of transcriptome complexity and regulation through the lens of local splicing variations. *elife* **5**, e11752 (2016).

[7] Heidelberger, P. & Welch, P. D. Simulation run length control in the presence of an initial transient. *Operations Research* **31**, 1109–1144 (1983).

[8] Li, H., Han, D., Hou, Y., Chen, H. & Chen, Z. Statistical inference methods for two crossing survival curves: a comparison of methods. *PLoS One* **10**, e0116774 (2015).
