# Supplementary material for: A Bayesian model for unsupervised detection of RNA splicing based subtypes in cancers
Source: Nat Commun. 2023 Jan 4;14:63. doi: 10.1038/s41467-022-35369-0 (PMC9813260; doi:10.1038/s41467-022-35369-0)
Supplement: Supplementary file 3 — Description of Additional Supplementary Files [file 41467_2022_35369_MOESM3_ESM.pdf]

Description of Additional Supplementary Data

Supplementary Data 1: beatAML

Format defined above for the beatAML dataset.

Supplementary Data 2: beatAML Recursive Step 1

Format defined above for the first recursive step applied to the beatAML dataset.

Supplementary Data 3: beatAML AML Genes

Format defined above for the beatAML dataset using only AML related genes.

Supplementary Data 4: Drug p-values

Kruskal-Wallis p-values for differential drug response (measured as AUC) between the clusters discovered in the beatAML dataset using AML genes.

Supplementary Data 5: TARGET AML

Format defined above for the joined dataset of beatAML and TARGET pediatric AML datatsets.

Supplementary Data 6: TARGET B-ALL

Format defined above for the TARGET B-ALL datase
