# Supplementary material for: A Bayesian model for unsupervised detection of RNA splicing based subtypes in cancers
Source: Nat Commun. 2023 Jan 4;14:63. doi: 10.1038/s41467-022-35369-0 (PMC9813260; doi:10.1038/s41467-022-35369-0)
Supplement: Supplementary file 10 — Reporting Summary [file 41467_2022_35369_MOESM10_ESM.pdf]

nature research

Corresponding author(s): Yoseph Barash

Last updated by author(s): Oct 19, 2022

Reporting Summary

Nature Research wishes to improve the reproducibility of the work that we publish. This form provides structure for consistency and transparency in reporting. For further information on Nature Research policies, see our Editorial Policies and the Editorial Policy Checklist.

Statistics

For all statistical analyses, confirm that the following items are present in the figure legend, table legend, main text, or Methods section.

n/a	Confirmed	
☐	☒	The exact sample size (n) for each experimental group/condition, given as a discrete number and unit of measurement
☒	☐	A statement on whether measurements were taken from distinct samples or whether the same sample was measured repeatedly
☐	☒	The statistical test(s) used AND whether they are one- or two-sided<br>*Only common tests should be described solely by name; describe more complex techniques in the Methods section.*
☒	☐	A description of all covariates tested
☐	☒	A description of any assumptions or corrections, such as tests of normality and adjustment for multiple comparisons
☐	☒	A full description of the statistical parameters including central tendency (e.g. means) or other basic estimates (e.g. regression coefficient) AND variation (e.g. standard deviation) or associated estimates of uncertainty (e.g. confidence intervals)
☐	☒	For null hypothesis testing, the test statistic (e.g. F, t, r) with confidence intervals, effect sizes, degrees of freedom and P value noted<br>*Give P values as exact values whenever suitable.*
☐	☒	For Bayesian analysis, information on the choice of priors and Markov chain Monte Carlo settings
☒	☐	For hierarchical and complex designs, identification of the appropriate level for tests and full reporting of outcomes
☐	☒	Estimates of effect sizes (e.g. Cohen's d, Pearson's r), indicating how they were calculated

Our web collection on statistics for biologists contains articles on many of the points above.

Software and code

Policy information about availability of computer code

Data collection: No software was used to collect data.

Data analysis:
CHESSBOARD (v1.0) software and dependencies:
- python_version >= "3.8"
- pandas >= 1.1.5
- scipy >= 1.5.4
- numpy >= 1.19.1
- seaborn >= 0.11.1
- statsmodels >= 0.12.2
- scikit-learn >= 0.21.3
- matplotlib >= 3.3.4

GAMBIT (v1.0): Webtool for visualizing CHESSBOARD output
Ingenuity Pathway Analysis (IPA) software (v01-21-03): Software for gene set enrichment analysis
MAJIQ and VIOLA (v2.1.0): Splicing quantification and visualization software.
STAR (v2.5.2a): RNASeq alignment software. All RNA-seq data was aligned to ensembl GRCh38 transcriptome release v94.
MOCCASIN (v0.25): Splicing batch correction tool.

For manuscripts utilizing custom algorithms or software that are central to the research but not yet described in published literature, software must be made available to editors and reviewers. We strongly encourage code deposition in a community repository (e.g. GitHub). See the Nature Research guidelines for submitting code & software for further information.

Data

Policy information about availability of data

All manuscripts must include a data availability statement. This statement should provide the following information, where applicable:

- Accession codes, unique identifiers, or web links for publicly available datasets
- A list of figures that have associated raw data
- A description of any restrictions on data availability

The beatAML dataset can be accessed through the National Cancer Institute (NCI) at https://www.cancer.gov/about-nci/organization/ccg/blog/2019/beataml. The Therapeutically Applicable Research to Generate Effective Treatments (TARGET) dataset, phs000218, managed by the NCI can be accessed at www.ncbi.nlm.nih.gov/projects/gap/cgi-bin/study.cgi?study_id=phs000218.v22.p8. Information about TARGET can be found at http://ocg.cancer.gov/programs/target. The Penn HTSC dataset is available at GEO (GSE142514). The ENCODE knockout and eCLIP datasets from Van Nostrand et al. 2020 are available at https://www.encodeproject.org. The GTEx v7 whole blood data is available at \url{https://www.gtexportal.org/home/datasets}. All processed datasets are available in the Zenodo repository associated with this publication at https://zenodo.org/record/7245323#.Y1apPFLMKQc. The data generated in this study including algorithm output and data used to figures is described in the Supplementary Information and Source Data files and can be accessed in the Zenodo repository.

Field-specific reporting

Please select the one below that is the best fit for your research. If you are not sure, read the appropriate sections before making your selection.

☒ Life sciences ☐ Behavioural & social sciences ☐ Ecological, evolutionary & environmental sciences

For a reference copy of the document with all sections, see nature.com/documents/nr-reporting-summary-flat.pdf

Life sciences study design

All studies must disclose on these points even when the disclosure is negative.

Sample size	All data derive from publicly available datasets and we use all available samples.
Data exclusions	No samples were excluded from experiments.
Replication	Data and associated scripts to replicate all analyses are in the Zenodo repository at [XX]
Randomization	Samples were allocated to experiments based on provided disease labels.
Blinding	Blinding is not applicable because we only used datasets generated in other studies

Reporting for specific materials, systems and methods

We require information from authors about some types of materials, experimental systems and methods used in many studies. Here, indicate whether each material, system or method listed is relevant to your study. If you are not sure if a list item applies to your research, read the appropriate section before selecting a response.

Materials & experimental systems

n/a	Involved in the study	
☒	☐	Antibodies
☒	☐	Eukaryotic cell lines
☒	☐	Palaeontology and archaeology
☒	☐	Animals and other organisms
☒	☐	Human research participants
☒	☐	Clinical data
☒	☐	Dual use research of concern

Methods

n/a	Involved in the study	
☒	☐	ChIP-seq
☒	☐	Flow cytometry
☒	☐	MRI-based neuroimaging
